# Supplementary material for: The interactome of intact mitochondria by cross-linking mass spectrometry provides evidence for coexisting respiratory supercomplexes
Source: Mol Cell Proteomics. 2017 Dec 8;17(2):216–32. doi: 10.1074/mcp.RA117.000470 (PMC5795388; doi:10.1074/mcp.RA117.000470)
Supplement: Supplemental Data [file supp_RA117.000470_133922_0_supp_23978_fzffwf.zip › spectra_annotation/mito_spectra_annotation/242-1-2-1-4-1.pdf]

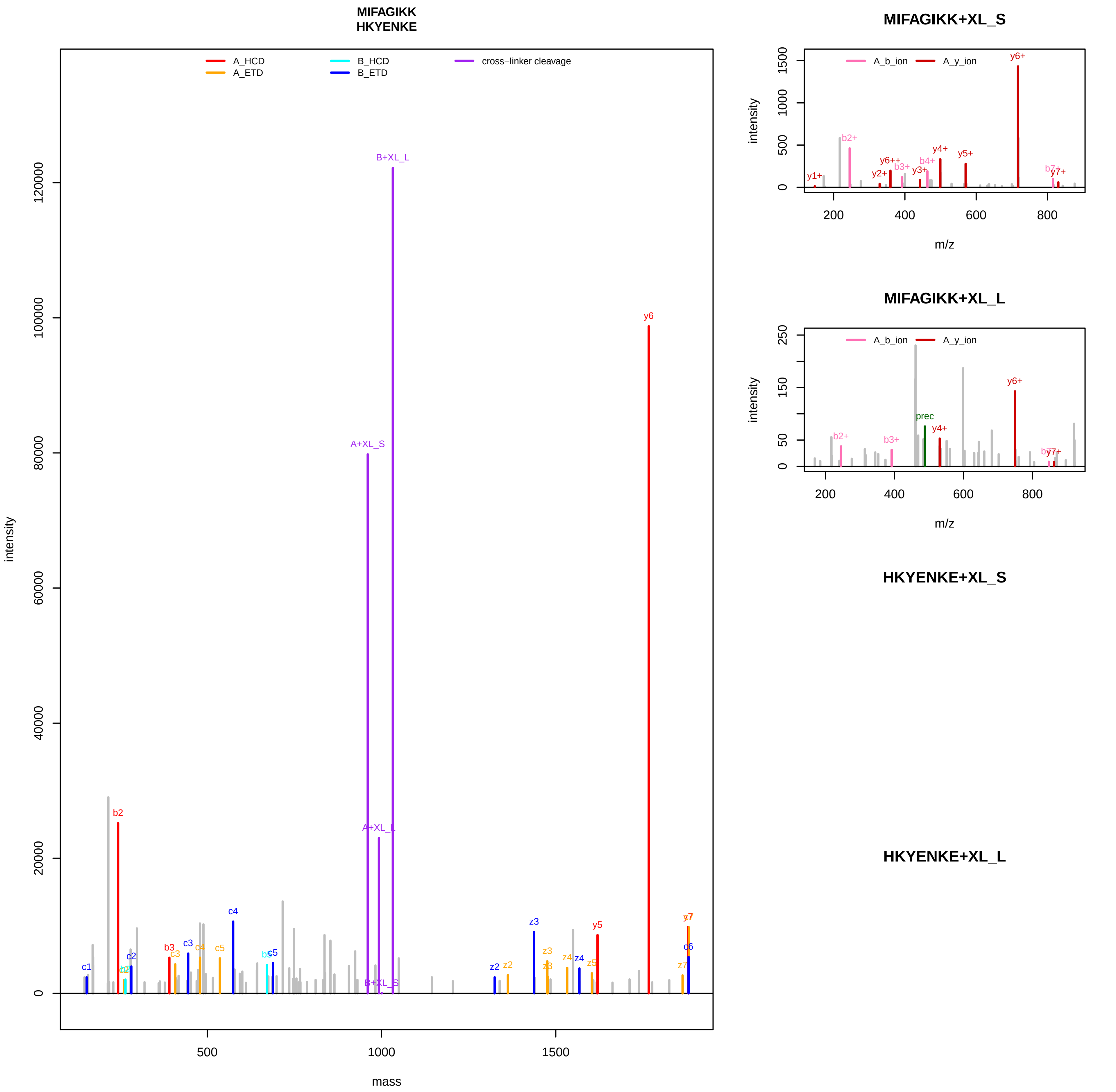
MIFAGIKK
HKYENKE
A_HCD
A_ETD
B_HCD
B_ETD
cross−linker cleavage
B+XL_L
A+XL_S
A+XL_L
B+XL_S
y6
y5
y7
z7
b2
b3
c1
c2
c3
c4
c5
c6
b5
z2
z3
z4
z5
intensity
mass
0
20000
40000
60000
80000
100000
120000
500
1000
1500
MIFAGIKK+XL_S
A_b_ion
A_y_ion
y1+
b2+
y2+
y6++
b3+
y3+
b4+
y4+
y5+
y6+
b7+
y7+
intensity
m/z
MIFAGIKK+XL_L
A_b_ion
A_y_ion
b2+
b3+
prec
y4+
y6+
b7+
y7+
intensity
m/z
HKYENKE+XL_S
HKYENKE+XL_L
